# P-2077. Pneumonia Outcomes in English Proficient vs Limited English Proficiency Patients at Jefferson Health New Jersey

**DOI:** 10.1093/ofid/ofaf695.2241

**Published:** 2026-01-11

**Authors:** Kristine Wong, Cindy M Hou, Toni Campanella, Venkat Venkataraman

**Affiliations:** Jefferson Health New Jersey, Stratford, NJ; Jefferson Health New Jersey, Stratford, NJ; Jefferson Health New Jersey, Stratford, NJ; Rowan-Virtua SOM, Startford, New Jersey

## Abstract

**Background:**

Language barriers contribute to impaired access to healthcare and are associated with poorer healthcare outcomes in the United States. Limited English proficiency is defined as having a primary language that is not English along with difficulty communicating in English. This study aims to determine if there is a difference in pneumonia outcomes between English proficient vs limited English proficiency (LEP) patient populations at Jefferson Health New Jersey.

**Figure:**
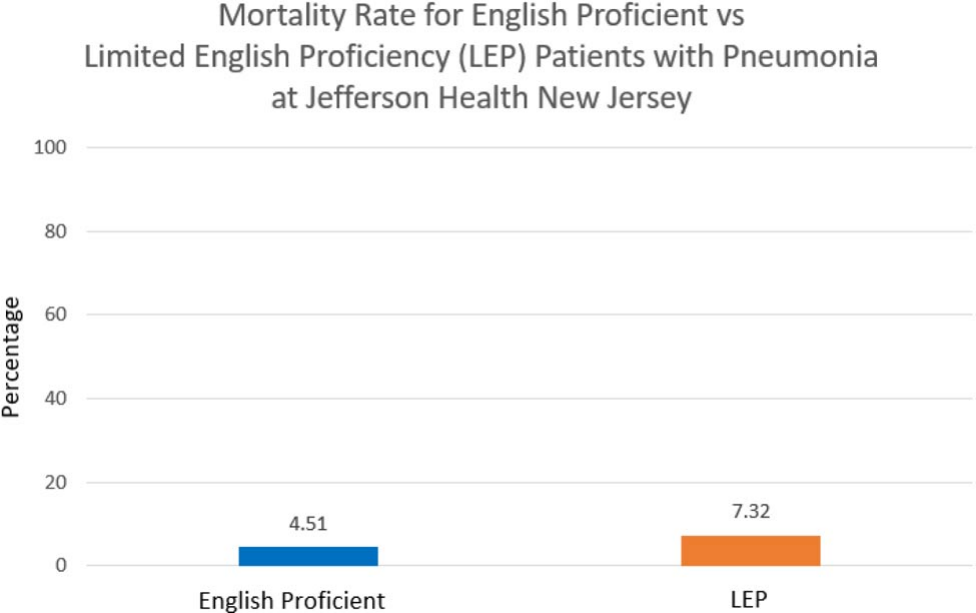

**Figure:**
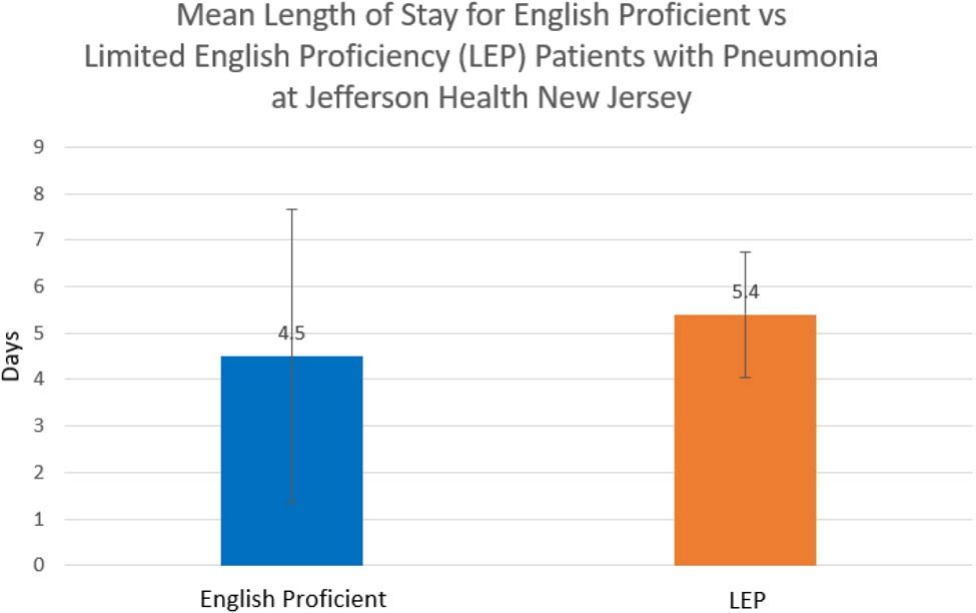

**Methods:**

This is a retrospective study using hospital admissions data from the three hospitals in the Jefferson Health New Jersey system - Jefferson Washington Township, Jefferson Cherry Hill, and Jefferson Stratford - from 6/27/23 to 5/31/24. A total of 928 patients were included in this study; 887 patients were in the English proficient group and 41 patients were in the LEP group. Inclusion criteria included patients at least 18 years of age or greater with a primary diagnosis of pneumonia. Categorization of patients into English proficient or LEP groups was based on self-reported designations as recorded in the electronic medical record. The primary endpoint was mortality and the secondary endpoint was mean length of stay.

**Results:**

The total mortality in English proficient patients was 42 deaths versus 3 deaths in LEP patients. Chi-square analysis to compare the difference in mortality resulted in a p-value of 0.45. Mean length of stay for English proficient patients was 4.50 days with a standard deviation of 4.00 days versus 5.40 days with a standard deviation of 7.20 days for LEP patients. The difference between the two means was 0.90 days and the 95% confidence interval for the difference between the two means was [-3.15, 1.35].

**Conclusion:**

The results of this study indicate that there is no significant difference in neither mortality nor mean length of stay between English vs LEP patient populations with the primary diagnosis of pneumonia at Jefferson Health New Jersey. This is significant because it suggests that there is no deficit in care based on English language proficiency when it comes to treatment for pneumonia.

**Disclosures:**

Cindy M. Hou, DO, MA, MBA, FACOI FIDSA, Abbott: Advisor/Consultant|Elsevier: royalty from COVID-19 book|Sanofi: Honoraria|Sepsis Alliance's PCORI Project: Advisor/Consultant

